# *N*-Phenyl­nicotinamide

**DOI:** 10.1107/S1600536809004863

**Published:** 2009-02-21

**Authors:** S. Mohana Roopan, Venkatesha R. Hathwar, A. Sudheer Kumar, N. Malathi, F. Nawaz Khan

**Affiliations:** a Chemistry Division, School of Science and Humanities, VIT University, Vellore 632 014, Tamil Nadu, India; b Solid State and Structural Chemistry Unit, Indian Institute of Science, Bangalore 560 012, Karnataka, India

## Abstract

In the title compound, C_12_H_10_N_2_O, the dihedral angle between the phenyl and pyridine rings is 64.81 (1)°. Inter­molecular N—H⋯O hydrogen bonds connect the mol­ecules into chains running along the *b* axis.

## Related literature

For general background, see: de Souza *et al.* (2005 ▶); Gdaniec *et al.* (1979 ▶). For related crystal structures, see: Cuffini *et al.* (2006 ▶). For graph-set motifs, see Bernstein *et al.* (1995 ▶).
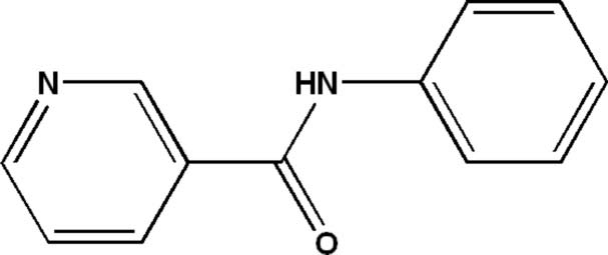


## Experimental

### 

#### Crystal data

C_12_H_10_N_2_O
*M* *_r_* = 198.22
Monoclinic, 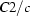

*a* = 18.732 (4) Å
*b* = 5.2766 (11) Å
*c* = 20.248 (4) Å
β = 103.746 (4)°
*V* = 1944.0 (7) Å^3^
*Z* = 8
Mo *K*α radiation
μ = 0.09 mm^−1^
*T* = 290 K
0.23 × 0.15 × 0.11 mm

#### Data collection

Bruker SMART CCD area-detector diffractometer
Absorption correction: multi-scan (*SADABS*; Sheldrick, 1996 ▶) *T*_min_ = 0.917, *T*_max_ = 0.990
6923 measured reflections
1813 independent reflections
1287 reflections with *I* > 2σ(*I*)
*R*_int_ = 0.039

#### Refinement

*R*[*F* ^2^ > 2σ(*F* ^2^)] = 0.049
*wR*(*F* ^2^) = 0.112
*S* = 1.03
1813 reflections
140 parameters
H atoms treated by a mixture of independent and constrained refinement
Δρ_max_ = 0.19 e Å^−3^
Δρ_min_ = −0.21 e Å^−3^

### 

Data collection: *SMART* (Bruker, 2004 ▶); cell refinement: *SAINT* (Bruker, 2004 ▶); data reduction: *SAINT*; program(s) used to solve structure: *SHELXS90* (Sheldrick, 2008 ▶); program(s) used to refine structure: *SHELXL97* (Sheldrick, 2008 ▶); molecular graphics: *ORTEP-3* (Farrugia, 1999 ▶) and *PLATON* (Spek, 2009 ▶); software used to prepare material for publication: *PLATON*.

## Supplementary Material

Crystal structure: contains datablocks global, I. DOI: 10.1107/S1600536809004863/bt2869sup1.cif

Structure factors: contains datablocks I. DOI: 10.1107/S1600536809004863/bt2869Isup2.hkl

Additional supplementary materials:  crystallographic information; 3D view; checkCIF report

## Figures and Tables

**Table 1:** Hydrogen-bond geometry (Å, °)

*D*—H⋯*A*	*D*—H	H⋯*A*	*D*⋯*A*	*D*—H⋯*A*
N2—H2N⋯O1^i^	0.91 (3)	2.26 (3)	3.088 (2)	152 (2)

Symmetry code: (i) 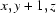.

**Fig. 1.**
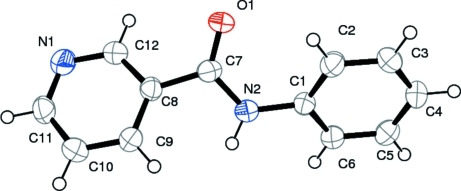
The molecular structure of the title compound shown with 50% probability displacement ellipsoids.

**Fig. 2.**
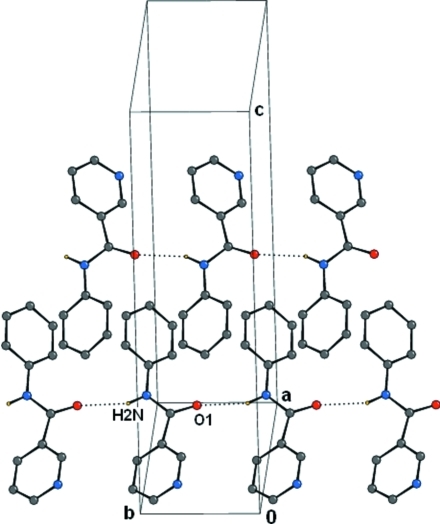
The crystal packing diagram of the title compound. The dotted lines indicate N—H···O intermolecular hydrogen bonds forming molecular chains along the b axis. All H atoms not involved in hydrogen bonds have been omitted for clarity.

## supplementary crystallographic information

### Comment

Nicotinamides were involved in biological processes such as production of energy, the synthesis of fatty acids, cholesterol and steroids, signal transduction, and the maintenance of the integrity of the genome (de Souza *et al.*, 2005). Nicotinamides play a major role in the prevention or delay of the onset of type 1 diabetes mellitus. They also have anti-oxidant, anti-inflammatory and anti-carcinogenic activities (Gdaniec *et al.*, 1979; Cuffini *et al.*, 2006).

The title compound is non planar molecule with a dihedral angle of 64.81 (1)° between the phenyl and pyridine ring. N—H···O intermolecular hydrogen bonds connect the molecules to one dimensional molecular chains along the *b* axis and forming a *C*(4) graph-set motif (Bernstein *et al.*, 1995).

### Experimental

Nicotinoyl chloride and aniline in tetrahydrofuran solution was stirred for 8 h at ambient temperature in the presence of a catalytic quantity of triethylamine. The reaction mixture was neutralized with a saturated aqueous sodium hydrogencarbonate solution and the resulting aqueous mixture was extracted with ethyl acetate and then concentrated under reduced pressure. Then, it was subjected to chromatography on silica, using hexane–ethyl acetate gradients. Crystals were grown from an ethanolic solution.

### Refinement

H atoms bonded to C were positioned geometrically and refined using a riding model with C—H bond lengths of 0.93 Å and *U*_iso_(H) = 1.2*U*_eq_(C). The H atom bonded to N was located from a difference Fourier map and refined isotropically.

### Crystal data

**Table:**

C_12_H_10_N_2_O	*F*(000) = 832
*M**_r_* = 198.22	*D*_x_ = 1.355 Mg m^−^^3^
Monoclinic, *C*2/*c*	Mo *K*α radiation, λ = 0.71073 Å
Hall symbol: -C 2yc	Cell parameters from 829 reflections
*a* = 18.732 (4) Å	θ = 2.0–24.4°
*b* = 5.2766 (11) Å	µ = 0.09 mm^−^^1^
*c* = 20.248 (4) Å	*T* = 290 K
β = 103.746 (4)°	Cylindrical, colourless
*V* = 1944.0 (7) Å^3^	0.23 × 0.15 × 0.11 mm
*Z* = 8	

### Data collection

**Table:**

Bruker SMART CCD area-detector diffractometer	1813 independent reflections
Radiation source: fine-focus sealed tube	1287 reflections with *I* > 2σ(*I*)
graphite	*R*_int_ = 0.039
φ and ω scans	θ_max_ = 25.5°, θ_min_ = 2.1°
Absorption correction: multi-scan (*SADABS*; Sheldrick, 1996)	*h* = −22→21
*T*_min_ = 0.917, *T*_max_ = 0.990	*k* = −6→6
6923 measured reflections	*l* = −24→24

### Refinement

**Table:**

Refinement on *F*^2^	Primary atom site location: structure-invariant direct methods
Least-squares matrix: full	Secondary atom site location: difference Fourier map
*R*[*F*^2^ > 2σ(*F*^2^)] = 0.049	Hydrogen site location: inferred from neighbouring sites
*wR*(*F*^2^) = 0.112	H atoms treated by a mixture of independent and constrained refinement
*S* = 1.03	*w* = 1/[σ^2^(*F*_o_^2^) + (0.0469*P*)^2^ + 0.7301*P*] where *P* = (*F*_o_^2^ + 2*F*_c_^2^)/3
1813 reflections	(Δ/σ)_max_ < 0.001
140 parameters	Δρ_max_ = 0.19 e Å^−^^3^
0 restraints	Δρ_min_ = −0.21 e Å^−^^3^

### Special details

**Table:**

Geometry. All e.s.d.'s (except the e.s.d. in the dihedral angle between two l.s. planes) are estimated using the full covariance matrix. The cell e.s.d.'s are taken into account individually in the estimation of e.s.d.'s in distances, angles and torsion angles; correlations between e.s.d.'s in cell parameters are only used when they are defined by crystal symmetry. An approximate (isotropic) treatment of cell e.s.d.'s is used for estimating e.s.d.'s involving l.s. planes.
Refinement. Refinement of *F*^2^ against ALL reflections. The weighted *R*-factor *wR* and goodness of fit *S* are based on *F*^2^, conventional *R*-factors *R* are based on *F*, with *F* set to zero for negative *F*^2^. The threshold expression of *F*^2^ > σ(*F*^2^) is used only for calculating *R*-factors(gt) *etc*. and is not relevant to the choice of reflections for refinement. *R*-factors based on *F*^2^ are statistically about twice as large as those based on *F*, and *R*- factors based on ALL data will be even larger.

### Fractional atomic coordinates and isotropic or equivalent isotropic displacement parameters (Å^2^)

**Table:**

	*x*	*y*	*z*	*U*_iso_*/*U*_eq_	
O1	0.70535 (8)	0.6222 (2)	0.07461 (7)	0.0535 (5)	
N1	0.50992 (9)	0.7693 (3)	−0.06750 (9)	0.0510 (5)	
N2	0.72283 (9)	1.0437 (3)	0.09527 (8)	0.0403 (4)	
H2N	0.7016 (12)	1.196 (5)	0.0809 (11)	0.075 (8)*	
C1	0.78193 (10)	1.0299 (3)	0.15450 (9)	0.0343 (5)	
C2	0.83422 (11)	0.8400 (4)	0.16449 (10)	0.0430 (5)	
H2	0.8316	0.7127	0.1322	0.052*	
C3	0.89028 (11)	0.8406 (4)	0.22253 (10)	0.0480 (6)	
H3	0.9255	0.7128	0.2292	0.058*	
C4	0.89503 (12)	1.0271 (4)	0.27086 (11)	0.0480 (6)	
H4	0.9327	1.0250	0.3102	0.058*	
C5	0.84308 (11)	1.2170 (4)	0.26008 (10)	0.0465 (6)	
H5	0.8460	1.3451	0.2922	0.056*	
C6	0.78691 (11)	1.2189 (4)	0.20234 (9)	0.0403 (5)	
H6	0.7522	1.3482	0.1955	0.048*	
C7	0.68817 (11)	0.8434 (3)	0.05987 (9)	0.0380 (5)	
C8	0.62589 (10)	0.9088 (3)	0.00086 (9)	0.0342 (5)	
C9	0.62425 (11)	1.1201 (3)	−0.03994 (9)	0.0392 (5)	
H9	0.6620	1.2387	−0.0303	0.047*	
C10	0.56564 (11)	1.1513 (4)	−0.09499 (10)	0.0458 (5)	
H10	0.5633	1.2900	−0.1238	0.055*	
C11	0.51068 (12)	0.9730 (4)	−0.10646 (10)	0.0489 (6)	
H11	0.4714	0.9955	−0.1439	0.059*	
C12	0.56755 (11)	0.7414 (4)	−0.01519 (10)	0.0427 (5)	
H12	0.5687	0.6000	0.0125	0.051*	

### Atomic displacement parameters (Å^2^)

**Table:**

	*U*^11^	*U*^22^	*U*^33^	*U*^12^	*U*^13^	*U*^23^
O1	0.0640 (10)	0.0257 (7)	0.0580 (9)	0.0019 (7)	−0.0108 (8)	0.0037 (6)
N1	0.0437 (11)	0.0428 (10)	0.0586 (12)	−0.0019 (8)	−0.0036 (10)	−0.0025 (9)
N2	0.0454 (11)	0.0298 (9)	0.0410 (10)	0.0004 (8)	0.0012 (8)	−0.0012 (7)
C1	0.0331 (11)	0.0339 (10)	0.0336 (10)	−0.0032 (8)	0.0036 (9)	0.0021 (8)
C2	0.0419 (12)	0.0383 (11)	0.0455 (12)	−0.0003 (9)	0.0038 (10)	−0.0076 (9)
C3	0.0403 (12)	0.0385 (11)	0.0590 (14)	0.0078 (9)	−0.0006 (11)	0.0027 (10)
C4	0.0416 (13)	0.0482 (12)	0.0454 (12)	−0.0047 (10)	−0.0070 (10)	0.0019 (10)
C5	0.0513 (14)	0.0401 (12)	0.0437 (12)	−0.0049 (10)	0.0028 (11)	−0.0094 (9)
C6	0.0403 (12)	0.0344 (10)	0.0431 (12)	0.0012 (9)	0.0036 (10)	−0.0019 (9)
C7	0.0433 (12)	0.0308 (10)	0.0392 (11)	−0.0019 (9)	0.0083 (9)	−0.0010 (8)
C8	0.0347 (11)	0.0349 (10)	0.0318 (10)	0.0022 (9)	0.0052 (9)	−0.0020 (8)
C9	0.0383 (11)	0.0364 (10)	0.0403 (11)	−0.0026 (9)	0.0039 (10)	−0.0027 (9)
C10	0.0512 (14)	0.0365 (11)	0.0460 (12)	0.0057 (10)	0.0042 (11)	0.0048 (9)
C11	0.0449 (13)	0.0468 (12)	0.0456 (12)	0.0093 (10)	−0.0076 (10)	−0.0033 (10)
C12	0.0441 (13)	0.0342 (11)	0.0464 (12)	−0.0007 (9)	0.0038 (11)	0.0021 (9)

### Geometric parameters (Å, °)

**Table:**

O1—C7	1.228 (2)	C4—H4	0.9300
N1—C12	1.329 (2)	C5—C6	1.375 (3)
N1—C11	1.335 (3)	C5—H5	0.9300
N2—C7	1.354 (2)	C6—H6	0.9300
N2—C1	1.428 (2)	C7—C8	1.498 (3)
N2—H2N	0.91 (2)	C8—C12	1.382 (3)
C1—C6	1.378 (3)	C8—C9	1.384 (2)
C1—C2	1.382 (3)	C9—C10	1.376 (3)
C2—C3	1.377 (3)	C9—H9	0.9300
C2—H2	0.9300	C10—C11	1.373 (3)
C3—C4	1.376 (3)	C10—H10	0.9300
C3—H3	0.9300	C11—H11	0.9300
C4—C5	1.378 (3)	C12—H12	0.9300
			
C12—N1—C11	116.06 (18)	C5—C6—H6	119.9
C7—N2—C1	125.70 (17)	C1—C6—H6	119.9
C7—N2—H2N	113.6 (14)	O1—C7—N2	123.27 (18)
C1—N2—H2N	120.3 (14)	O1—C7—C8	121.46 (17)
C6—C1—C2	119.65 (17)	N2—C7—C8	115.27 (16)
C6—C1—N2	117.58 (17)	C12—C8—C9	118.05 (17)
C2—C1—N2	122.76 (17)	C12—C8—C7	117.55 (17)
C3—C2—C1	119.56 (18)	C9—C8—C7	124.36 (17)
C3—C2—H2	120.2	C10—C9—C8	118.71 (18)
C1—C2—H2	120.2	C10—C9—H9	120.6
C4—C3—C2	121.05 (19)	C8—C9—H9	120.6
C4—C3—H3	119.5	C11—C10—C9	118.47 (19)
C2—C3—H3	119.5	C11—C10—H10	120.8
C3—C4—C5	118.93 (19)	C9—C10—H10	120.8
C3—C4—H4	120.5	N1—C11—C10	124.37 (19)
C5—C4—H4	120.5	N1—C11—H11	117.8
C6—C5—C4	120.61 (19)	C10—C11—H11	117.8
C6—C5—H5	119.7	N1—C12—C8	124.33 (18)
C4—C5—H5	119.7	N1—C12—H12	117.8
C5—C6—C1	120.20 (18)	C8—C12—H12	117.8
			
C7—N2—C1—C6	−148.13 (19)	O1—C7—C8—C12	31.6 (3)
C7—N2—C1—C2	33.3 (3)	N2—C7—C8—C12	−147.87 (18)
C6—C1—C2—C3	0.7 (3)	O1—C7—C8—C9	−145.9 (2)
N2—C1—C2—C3	179.18 (18)	N2—C7—C8—C9	34.6 (3)
C1—C2—C3—C4	0.1 (3)	C12—C8—C9—C10	−1.2 (3)
C2—C3—C4—C5	−0.8 (3)	C7—C8—C9—C10	176.34 (18)
C3—C4—C5—C6	0.7 (3)	C8—C9—C10—C11	1.0 (3)
C4—C5—C6—C1	0.1 (3)	C12—N1—C11—C10	−1.0 (3)
C2—C1—C6—C5	−0.8 (3)	C9—C10—C11—N1	0.2 (3)
N2—C1—C6—C5	−179.35 (17)	C11—N1—C12—C8	0.8 (3)
C1—N2—C7—O1	−1.5 (3)	C9—C8—C12—N1	0.3 (3)
C1—N2—C7—C8	177.91 (17)	C7—C8—C12—N1	−177.39 (19)

### Hydrogen-bond geometry (Å, °)

**Table:**

*D*—H···*A*	*D*—H	H···*A*	*D*···*A*	*D*—H···*A*
N2—H2N···O1^i^	0.91 (3)	2.26 (3)	3.088 (2)	152 (2)

Symmetry codes: (i) *x*, *y*+1, *z*.

## References

[bb1] Bernstein, J., Davis, R. E., Shimoni, L. & Chang, N.-L. (1995). *Angew. Chem. Int. Ed. Engl.* **34**, 1555-1573.

[bb2] Bruker (2004). *SMART* and *SAINT* Bruker AXS Inc., Madison, Wisconsin, USA.

[bb3] Cuffini, S., Glidewell, C., Low, J. N., de Oliveira, A. G., de Souza, M. V. N., Vasconcelos, T. R. A., Wardell, S. M. S. V. & Wardell, J. L. (2006). *Acta Cryst.* B**62**, 651–665. 10.1107/S0108768106015497 16840815

[bb4] Farrugia, L. J. (1999). *J. Appl. Cryst.* **32**, 837–838.

[bb5] Gdaniec, M., Jaskolski, M. & Kosturkiewicz, Z. (1979). *Pol. J. Chem.* **53**, 2563–2569.

[bb6] Sheldrick, G. M. (1996). *SADABS* University of Göttingen, Germany.

[bb7] Sheldrick, G. M. (2008). *Acta Cryst.* A**64**, 112–122. 10.1107/S0108767307043930 18156677

[bb8] Souza, M. V. N. de, Vasconcelos, T. R. A., Wardell, S. M. S. V., Wardell, J. L., Low, J. N. & Glidewell, C. (2005). *Acta Cryst.* C**61**, o204–o208. 10.1107/S0108270105003586 15805626

[bb9] Spek, A. L. (2009). *Acta Cryst.* D**65**, 148–155. 10.1107/S090744490804362X PMC2631630 19171970

